# Optical Coherence Tomography Angiography versus Dye Angiography in Age-Related Macular Degeneration: Sensitivity and Specificity Analysis

**DOI:** 10.1155/2018/6724818

**Published:** 2018-03-07

**Authors:** Eleni Nikolopoulou, Massimo Lorusso, Luisa Micelli Ferrari, Maria Vittoria Cicinelli, Francesco Bandello, Giuseppe Querques, Tommaso Micelli Ferrari

**Affiliations:** ^1^Department of Ophthalmology, Ente Ecclesiastico Ospedale Generale Regionale “F. Miulli”, Acquaviva delle Fonti, Bari, Italy; ^2^Department of Ophthalmology, Vita-Salute University, San Raffaele Scientific Institute, Via Olgettina 60, 20132 Milan, Italy

## Abstract

**Introduction:**

Optical coherence tomography angiography (OCTA) could be a valid tool to detect choroidal neovascularization (CNV) in neovascular age-related macular degeneration (nAMD), allowing the analysis of the type, the morphology, and the extension of CNV in most of the cases.

**Purpose:**

To determine the sensitivity and specificity of OCTA in detecting CNV secondary to nAMD, compared to fluorescein angiography (FA) and indocyanine green angiography (ICGA).

**Methods:**

Prospective observational study. Patients with suspected nAMD were recruited between May and December 2016. Patients underwent FA, ICGA, spectral domain OCT, and OCTA (AngioVue, Optovue, Inc.). Sensitivity and specificity of FA, with or without ICGA, were assessed and compared with OCTA.

**Results:**

Seventy eyes of 70 consecutive patients were included: 32 eyes (45.7%) with type I CNV, 8 eyes (11.4%) with type II CNV, 4 eyes (5.7%) with type III CNV, 6 eyes (8.6%) with mixed type I and type II CNV, and 20 eyes (28.6%) with no CNV. Sensitivity of OCTA was 88% and specificity was 90%. Concordance between FA/ICGA and OCTA was very good (0,91; range 0,81–1,00).

**Conclusions:**

OCTA showed high sensitivity and specificity for detection of CNV. Concordance between OCTA and gold-standard dye-based techniques was excellent. OCTA may represent a first-line noninvasive method for the diagnosis of nAMD.

## 1. Introduction

Age-related macular degeneration (AMD) is the leading cause of severe and irreversible central visual loss in developed countries, affecting 10%–13% of adults over 65 years of age [1]. The introduction of antivascular endothelial growth factor (VEGF) therapy has changed the natural history of neovascular AMD (nAMD) [2, 3]. Individuals aged more than 50 years, with high-risk factors, like family history of AMD and history of smoking, should undergo AMD screening on regular bases, to early detect the presence of choroidal neovascularization (CNV) in the setting of nAMD and give the appropriate treatment.

Screening methods include visual acuity measurement and fundus examination, combined with self-monitoring Amsler grid [4]. In cases of suspected nAMD, further diagnostic testing should be performed, as fluorescein angiography (FA), indocyanine green angiography (ICGA), and optical coherence tomography (OCT). FA provides anatomical details, detects the grade of activity, and plays a key role in the classification of the CNV [5]. The neovascular network of occult CNV is better identified by ICGA [6, 7]. ICGA is also useful in the diagnosis of two other specific forms of nAMD, retinal angiomatous proliferation (RAP) and polypoidal choroidal vasculopathy (PCV), characterized by lower incidence, more aggressive natural history, and poorer response to antiangiogenic therapy [8, 9].

Spectral domain OCT (SD-OCT) is a noninvasive imaging technique, able to visualize structural changes of the neurosensory retina and the retinal pigment epithelium (RPE). OCT is used to support the initial diagnosis of CNV obtained from FA and ICGA and to detect early signs of CNV activity, such as subretinal fluid, intraretinal cystoid spaces, and pigment epithelium detachment (PED) [10, 11].

Recently, OCT angiography (OCTA) has been introduced in the clinical practice. OCTA provides cross-sectional and three-dimensional imaging of the retinal and choroidal vasculature with micrometer-scale depth resolution [12, 13]. The potential role of OCTA in the first noninvasive diagnosis of nAMD, combined with or without gold-standard dye angiographic techniques, is still object of debate.

The aim of the present study is to estimate the sensitivity and specificity of OCTA in assessing the presence of CNV in nAMD, compared to gold-standard techniques (FA and ICGA).

## 2. Materials and Methods

This clinical cross-sectional study was conducted in the Department of Ophthalmology, Miulli Hospital, Acquaviva delle Fonti, Italy. The study adhered to the tenets of the Declaration of Helsinki and patients signed written consent before being included in the study.

Data of patients presenting at the Retina Service between May and December 2016 were analyzed. We included both treatment-naïve patients and those already treated with intravitreal anti-VEGF. Inclusion criteria were the presence of active CNV secondary to nAMD, age ≥ 50 years, sufficiently clear ocular media, and adequate pupillary dilation and fixation to permit high-quality imaging. The diagnosis of active CNV was based on combining FA and OCT examination. Exclusion criteria were the presence of CNV secondary to retinal diseases other than AMD, along with allergy or medical contraindication to intravenous dyes (fluorescein and indocyanine green). In eyes presenting with bilateral exudative AMD, the eye with the most clinically active disease was arbitrarily chosen.

Each patient underwent a comprehensive ophthalmologic examination, including measurement of best-corrected visual acuity (BCVA), dilated slit-lamp biomicroscopy, FA and ICGA (Spectralis + HRA; Heidelberg Engineering, Heidelberg, Germany), SD-OCT, and OCTA (AngioVue, Optovue, Inc.) in the same visit. On the basis of imaging results, the CNV, if present, was classified as follows: type I, presenting occult leakage on FA and neovascular complex below RPE on OCT B-scan; type II, showing classic well-localized leakage on FA and neovascular lesion above RPE on OCT B-scan; and type III, presenting focal leakage on FA and retinochoroidal vascular anastomosis on OCT B-scan.

The OCTA was performed using the AngioVue System, XR Avanti SD-OCT device (Optovue, Inc., Fremont, CA, USA), based on a high-speed SD-OCT platform that operates at 70,000 axial scans per second. Each B-scan in the OCT volume consists of 304 A-scans and is repeated twice at the same retinal location. OCTA scan size of 3 × 3 mm was chosen for our purposes; in case of partial visualization of the entire CNV network, a larger (6 × 6 mm) image was obtained. Scans with low quality (i.e., if the subject blinked, in case of unstable fixation, or if there were many motion artifacts in the data set) were excluded and repeated until good quality was achieved. The AngioVue System offers automatic segmentation of the retinal and choroidal layers; each layer is displayed as the en-face angiogram and coregistered with the cross-sectional OCT B-scans. The software allows manually changing the depth and width of the inner and outer boundary lines, to better visualize CNV plane.

Two trained graders (E.N. and M.L.) independently evaluated FA, IGCA, and OCTA scan separately for each patient. The detection of CNV on FA was assessed by the presence of late phase leakage (5 min); ICGA was considered positive if a neovascular complex was visualized on early phase (0-1 min) or if a plaque or hot spot was seen on late phase (15–20 min). OCTA was considered positive if an abnormal flow signal was seen in the outer retina or in the choriocapillaris.

The sensitivity and the specificity of OCTA for neovascular detection (i.e., new diagnostic tool validity assessment) were estimated in comparison to FA, considered as the gold-standard. An additional cohort of patients with nonexudative AMD was enrolled as negative controls. Nonexudative AMD was defined as presence of drusen or RPE changes in the macula, with no evidence of vascular network on FA or ICGA; patients with advanced form of AMD (geographic atrophy) were not included. If an eye was determined to have a CNV on FA, an OCTA showing an abnormal neovascular network was considered a true positive; if the CNV was not visualized on OCTA, the examination was taken as a false negative. If the FA did not demonstrate a CNV, an OCTA with no evidence of CNV was considered as a true negative; if a CNV was detected, the examination was considered a false positive.

To examine the concordance between FA and OCTA in assessing the presence CNV, the interrater agreement k-analysis (Cohen's kappa coefficient) was used. The analysis was repeated combining FA with ICGA. The calculation of the *κ*-value with its 95% confidence interval was performed. The kappa-statistic was interpreted according to the classification provided by Landis and Koch and specifically as follows: <0, poor agreement; 0–0.20, slight agreement; 0.21–0.40, fair agreement; 0.41–0.60, moderate agreement; 0.61–0.80, substantial agreement; >0.80, almost perfect agreement [14].

Statistical analysis, including descriptive statistics for demographics and main clinical records, was performed through GraphPad Prism V.6.0 (GraphPad software, San Diego, California, USA).

## 3. Results

We included 70 consecutive patients affected by AMD. All patients were Caucasian; mean age was 70.9 ± 10.27 years (Table 1). According to FA and SD-OCT, 50 eyes were affected by nAMD; 32 eyes (45.7%) featured type I CNV; 8 eyes (11.4%) featured type II CNV; 4 eyes (5.7%) featured type III CNV; 6 eyes (8.6%) featured mixed type I + II CNV (Table 1). Twenty eyes with nonexudative AMD acted as negative controls. Among those with nAMD, 17 patients had previously received anti-VEGF treatment; the remaining 33 eyes were treatment-naïve. No adverse event related to the diagnostic procedures was reported.

**Figure 1:**
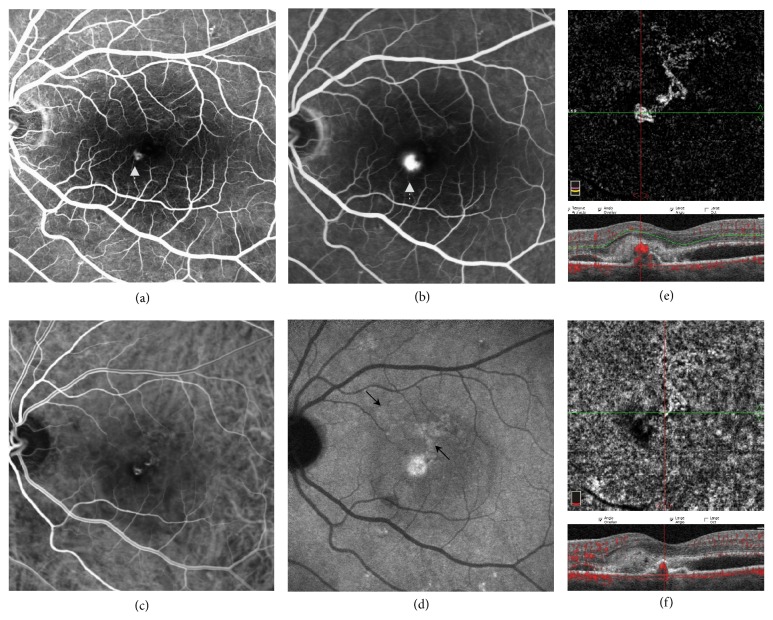
Multimodal imaging of a type II choroidal neovascularization (CNV), evaluated as a true positive case. (a-b) Early- and late-frame fluorescein angiography displaying a small area of early and late leakage (white arrow) in the juxtafoveal area. (c-d) Early- and late-frame indocyanine green angiography showing the presence of two small hyperfluorescent areas featuring late leakage (black arrows). (e-f) A 3 × 3 mm optical coherence tomography angiography slab at the outer retina and at the choriocapillaris showing a well-circumscribed branched CNV.

**Figure 2:**
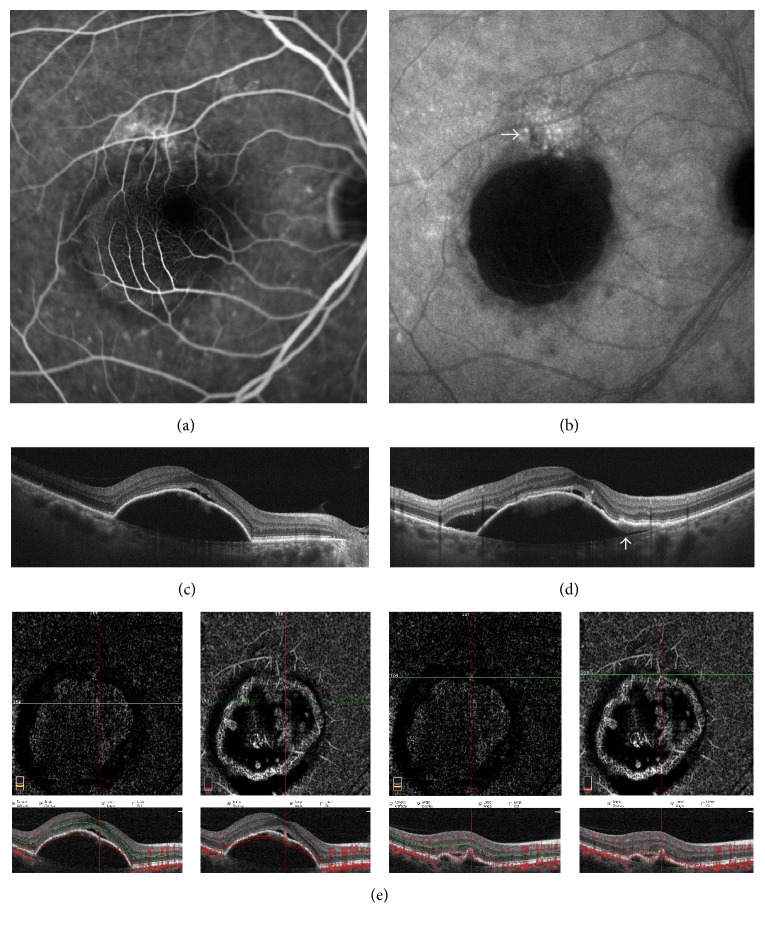
Multimodal imaging of a false negative case. (a) Late-frame fluorescein angiography displaying a small area of dye leakage superiorly to a central area of hypofluorescence. (b) Late-frame indocyanine green angiography confirms the presence of a neovascular plaque superiorly to the central area of deep hypocyanescence. (c-d) B-scan optical coherence tomography demonstrates a high serous pigment epithelium detachment (PED) with evidence of a poorly reflective tissue under the retinal pigment epithelium (white arrow) and subretinal fluid. A small rip of the retinal pigment epithelium could be suspected (e) OCT angiography 6 × 6 scans: evidence of flow signal neither at the outer retina layer nor at the choriocapillaris layer due to the masking artifact of the PED.

**Figure 3:**
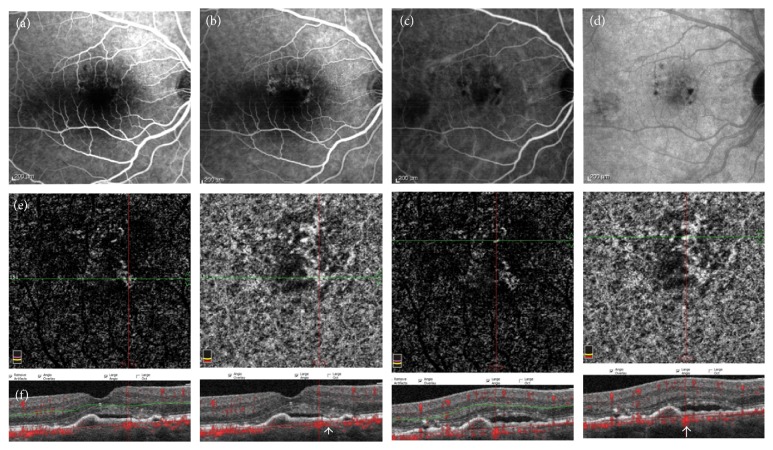
Multimodal imaging of a false-positive case. (a-b) Fluorescein angiography displaying late hyperfluorescence superiorly and nasally to the fovea. (c-d) Indocyanine green angiography showing no neovascular plaque. (e) 3 × 3 mm optical coherence tomography angiography (OCTA) with evidence of an ill-defined neovascular network at the outer retina and choriocapillaris layer. (f) Coregistered OCT B-scans show subfoveal pigment epithelium detachment (PED) and subretinal fluid; on cross-sectional scans flow signal under the PED is evident (white arrow).

On OCTA, a CNV was recognized in 46 eyes; 44 were true positives (Figure 1). Six cases were evaluated as false negatives (Figure 2). In 18 of the 20 controls, both OCTA and FA did not identify any CNV; in the 2 remaining controls, the OCTA was positive while FA was negative (Table 2) (Figure 3). The sensitivity of OCTA was 88% and the specificity was 90%. CNV appeared on OCTA as either a main feeder vessel with numerous secondary anastomotic vessels or a large central trunk with poorly organized vascular branches.

Concordance between OCTA and FA was good, and Cohen's kappa coefficient was 0.88 (0.81–1.00). Combining ICGA to FA, kappa coefficient was 0.91 (0.81–1.0) (Table 3). Concordance was slightly lower when only CNV type I was included in the analysis.

## 4. Discussion

Our study aimed to investigate the capability of the noninvasive dye-less OCTA technique in detecting CNV, compared to traditional dye angiography (FA and ICGA). In our study, OCTA demonstrated high ability to identify and visualize the neovascular complex, in accordance with FA. On the contrary, OCTA did not provide any evidence of CNV, depicted on FA, in 9% of eyes (6 eyes). False negatives presented a high serous-hemorrhagic PED, which limited the penetration of the OCTA signal under the RPE. In addition, the distortion of the retinal architecture caused by the RPE elevation creates difficulty in identifying the underlying neovascular complex, even after manual adjustment of the segmentation lines. True negative cases (18 eyes, 26%) featured drusenoid or serous PED without any associated occult CNV.

A small number of cases in our series (two eyes, 3%) were evaluated as false positives, as the OCTA demonstrated the presence of a neovascular complex in the choriocapillaris layer, despite negative FA and ICGA. In one of the two patients, the OCT B-scan presented a flat PED with no sub- or intraretinal fluid. This clinical feature has been interpreted as quiescent CNV, as described by Querques et al. [15, 16]. In the second case, we can speculate that traditional angiography imaging would have been flawed by some masking artifacts, hiding the presence of a neovascular network (visualized instead on OCTA).

The sensitivity and specificity of OCTA in detecting the CNV secondary to nAMD have been investigated in different studies. At first, Jia et al. demonstrated the ability of OCTA to detect and quantify CNV in 10 subjects affected by nAMD [17]. According to the authors, OCTA provided better visualization of the neovascular network with respect to FA, as images were not obscured by subretinal hemorrhage or other artifacts. Our results were partially in discordance with Jia's conclusions, as serous-hemorrhagic PED limited the penetration of the OCTA signal in our study population. De Carlo and associates found that sensitivity and specificity of OCTA were 50% and 91%, respectively [18]. Upon review of their false negatives, 3 out of 4 cases had a large subretinal hemorrhage. Differently, the authors concluded that OCTA does not perfectly detect the extent of CNV in cases or large subretinal hemorrhages. A study conducted by Kuehlewein et al. on occult neovascular membranes in nAMD concluded that OCTA identified the vascular complex in 75% of cases [19]. Recently, Souedan and his group evaluated the diagnostic accuracy of OCTA in detecting CNV compared to FA only and FA coupled to SD-OCT, graded independently by ophthalmologists with varying expertise levels [20]. In this study, OCTA was more sensitive than FA alone. However, when FA was combined with SD-OCT it remained more sensitive and specific (sensitivity of 92.72% and specificity of 90.91%) than OCTA alone (sensitivity of 85.62% and specificity of 81.51%).

Our findings demonstrate that OCTA is able to detect the neovascular complex in most of the cases of nAMD, allowing the analysis of the morphology of the CNV in every single patient. According to the most recent literature, new vascular proliferation is usually characterized by well-defined complexes, with a main feeder vessel and numerous tiny anastomotic capillaries with thin walls and small diameter [21]. On the contrary, inveterate neovascular lesions, already treated with antiangiogenic therapy, show a thick central trunk with large anastomotic vessels, probably due to an arteriogenesis process during vessel branches expansion [21]. Our findings substantially agree with this features of CNV. We can speculate that OCTA would display worse sensitivity in naïve CNV, due to undetectable flow inside the small peripheral branches of the neovascular complex; further studies, specifically addressing naïve nAMD, are warranted.

The main novelty of the study is the calculation of agreement between OCTA and FA combined with ICGA, which was excellent in all the cases. A subanalysis, including only patients featuring type I CNV, showed a slightly worse correlation between the OCTA and the gold-standard. We can speculate that this is due to the presence of PED or RPE abnormalities in occult CNVs, which limited the correct visualization of the CNV under the RPE.

Limitations of the study are the small size of the sample and the heterogeneity of the included population. In fact, we considered patients with different types of CNV—most were type I CNV—and both naïve and treated patients. It would be interesting to repeat the same analysis by dividing the patients into separate groups.

## 5. Conclusion

Our findings demonstrated that OCTA is a valid tool to detect the neovascular complex in nAMD. In detail, OCTA allowed the diagnosis of CNVs in most of the cases analyzed, with the important exception of cases presenting subretinal hemorrhages or high serous-hemorrhagic PEDs. In routine practice, OCTA is still coupled with angiography for the diagnosis and the follow-up of nAMD. Further studies are necessary to understand the possibility of noninvasive OCTA to completely replace conventional dye tests, avoiding their unpredictable side effects.

## Figures and Tables

**Table 1:** Demographic and clinical data of the study patients.

Gender	
Female	28 (40%)
Male	42 (60%)
Ethnic origin	
Caucasian	70 (100%)
Age (years)	
Mean	70,8
SD	10,3
Min	51
Max	92
AMD classification	
Type I	32 (45.7%)
Type II	8 (11.4%)
Type III	4 (5.7%)
Type I + II	6 (8.6%)
No CNV	20 (28.6%)

SD= standard deviation; AMD= age-related macular degeneration; CNV= choroidal neovascularization. For CNV classification, see the text.

**Table 2:** 2 × 2 contingency table computing relative CNV detection rate between optical coherence tomography angiography and fluorescein angiography.

	FA		
	Positive	Negative	Total
OCTA			
Positive	44	2	46
Negative	6	18	24
Total	50	20	70

OCTA = optical coherence tomography angiography; FA = fluorescein angiography.

**Table 3:** Interrater agreement between the different instruments (Cohen's Kappa Index).

All CNV types	FA versus OCTA	0.88
	FA + ICGA versus OCTA	0.91
Only CNV type I	FA + ICGA versus OCTA	0.84

CNV = choroidal neovascularization; FA = fluorescein angiography; OCTA = optical coherence tomography angiography; ICGA = indocyanine green angiography.
